# The Addicted Brain

**DOI:** 10.3389/fpsyt.2013.00040

**Published:** 2013-06-03

**Authors:** Marco Diana

**Affiliations:** ^1^“G. Minardi” Laboratory of Cognitive Neuroscience, Department of Chemistry and Pharmacy, University of SassariSassari, Italy

## Defining Addiction

Classification of behavioral disturbances should be done by carefully considering the boundaries between physiology (normality) and pathology (disease), irrespective of social context. The following considerations will highlight some of the objectivable changes observed in the brains of animals and humans who underwent repetitive use of addicting chemicals. These molecular, cellular, and even structural, carefully documented and widely reproduced, changes, which take place at the level of the brain, form the basis for classifying addiction as a brain disease, *as defined in DSM IV-R*. Attributing to addiction the status of disease has several advantages and virtually no disadvantage. The first, major advantage is for the sufferer/addict himself: it confers automatically the status of patient and excludes another status, frequently attributed to him by societal custom: delinquent. Another advantage is that research on the biological basis of a widespread disease such as addiction will continue to be fostered by (desperately needed) public funding and, hopefully, efficient therapies will be found. Addiction is a brain disease which impairs behavior: the product of brain activity. As such should be classified, studied, treated, and hopefully cured. The whole society will benefit from it.

## Levy’s Approach

In his essay Levy (2013) approaches the problem of addiction (*without providing a definition*) in a mixed way providing a mostly sociological perspective that leads him to conclude that addiction is not a brain disease. I will argue that this is not the way the problem should be approached. I was taught early (as a medical student) in my life that a disease (pathology) is a derailment from normal functioning of a system/organ/cell (physiology), and ultimately of the whole organism. A frequently employed example is inflammation: perhaps the simplest form of pathology. It has well defined characteristics which, for many of the old continent, were described in latin: *rubor*, *tumor*, *calor*, *dolor*, *and functio lesa*. When these characteristics are present, the pathology/disease can be diagnosed. If it occurs in the lung it will indicate pneumonia, in the kidney nephritis etc. Likewise, a brain chronically receiving drugs changes its physiology, its biochemistry, and even its anatomy in its most intimate structure such as dendritic spines *and genes*. An excellent example (*but many other exist*) is provided by one of the most intensely studied neuronal systems of the brain in addiction studies: the dopamine system.

## Neurobiology

This system is profoundly hypofunctional after being targeted by chronic drugs of abuse (Melis et al., 2005): electrophysiological activity is reduced (Diana et al., 1993, 1995, 1998), release is hampered and cell bodies shrink (Spiga et al., 2003). On the postsynaptic side similar changes occur and spine density loss has been documented in amphetamine-experienced (Robinson and Kolb, 1997, 2004), alcohol-dependent (Diana et al., 2003), morphine-withdrawn (Spiga et al., 2005), and cannabis-*addicted* rodents (Spiga et al., 2010). Human studies (key to validate animal models) are equally informative and supportive of a compromised role of DA transmission in human addicts. For instance, while alcohol increases DA release in healthy subjects (Boileau et al., 2003) with some gender differences (Urban et al., 2010), a reduced number of DA receptors has been observed (Volkow et al., 1996; Martinez et al., 2005) in alcoholics that appears to be accompanied by a blunted DA release (Martinez et al., 2005, 2007; Volkow et al., 2007). While the reduced number of DA receptors could be, at first sight, be viewed as suggesting an augmented DA release, it should be noted that by administering the DA inhibitor alpha methyl-para-tyrosine, Martinez et al. (2009) were able to exclude this possibility. Indeed, while healthy controls do show an increased raclopride binding after acute alpha methyl-para-tyrosine administration, cocaine-dependent subjects do not (or to a significantly lesser extent). Similar results were obtained with the dopamine releasing agent methylphenidate (Volkow et al., 2007) and amphetamine (Martinez et al., 2005) in alcoholics. Notably, artificially increasing the brain levels of DAD2 receptors, using a replication-deficient adenoviral vector containing the rat cDNA insert for DAD2 into the Nacc, reduces alcohol intake in spontaneously drinking rats, thereby offering Popper-style evidence that a potentiation of DA transmission may have beneficial effects on alcohol-seeking and alcohol-taking (Thanos et al., 2001, 2004). In line with this conclusion, a spontaneous high number of DA D2 receptors has been shown to have a protective role in non-alcoholic members of alcoholic families (Volkow et al., 2006). These findings further support the notion that the number of DA receptors (and consequently DA transmission) inversely correlates with alcohol pathological drinking.

## Neural Plasticity

Among the various points Levy raises is: “*It will not help the defender of the brain disease account to add the other neural correlates associated with addiction into the mix. Consider the chronic deviation from reward set point suggested by* Koob and Le Moal (1997, 2008). *The allostatic state they postulate is the result of the brain adapting to drug ingestion. So long as the drug is reliably available*, *the person will suffer no ill-effects from this neuroadaptation. Rather*, *the anhedonic state from which individuals suffer is associated with chronic abstinence. Identifying the pathology with this unpleasant state entails*, *counter-intuitively*, *that the abstinent addict suffers from a pathology but the addict who is using does not*.”

First: intuition is not a form of learning that is neurobiologically recognized and thus what Levy finds “counterintuitive” may simply be due to his personal expectations instead than stemming from observation of objective experimental facts. The allostatic concept of Koob and Le Moal (2008) simplified, says that the addicted brain is “stiffer” and less prone to adapt to homeostasis (equilibrium). Familiarizing with the neural plasticity concept could help: in poor words, brain plasticity allows the system (the brain) to adapt to new situations which include the drug of abuse (tolerance). It adapts to the new situation by implementing mechanisms to reduce harm that may be produced by the intruder (drug) which result in a new physiological status. If the drug is now abruptly removed from the system, then the suffering becomes evident with a number of signs and symptoms which characterize the withdrawal syndrome. Almost paradoxically, but only at first sight, in the addicted individual the drug works as a trophic factor that helps the whole system to function. When the trophic factor is removed the disease is unveiled.

Second: as long as the drug of choice is available the addict will not suffer because the drug itself is the “cure” of his pathology. Examples of this fact have been known for decades in the field and exemplified by methadone for opiate-addicts, nicotine patches for tobacco-dependent individuals and GHB for alcoholics (Gallimberti et al., 1989; Biggio et al., 1992). The problem emerges when the drug is *NO longer* available and the withdrawal syndrome ensues from it. This should not be seen as a bizarre or a curious event but is actually the rule in many medical instances. For example, diabetics suffer because their pancreas does not produce insulin, whose absence (if not compensated exogenously) may bring the patient to hyperglycemic coma. Similarly, in the addict, homeostasis is reached when the drug is “on-board” and not when the drug is withdrawn. Actually, withdrawal is a key phase of the whole addicting cycle (see Koob and Volkow, 2010). Not only this is the phase in which the addict will manifest a full-blown discomfort but is also the only phase in which he will seek treatment and will admit, possibly, his pathological status. Further, let’s take for example *malaria* (*i.e*, *jungle fever*). It is well known that fever peaks every 3–4 days depending on the plasmodium strain. Should we then conclude that the patient has malaria only when his temperature rises every 3 or 4 days? Clearly, the patient is afflicted by malaria even when the fever is gone and body temperature is normal.

In the case of addiction, its inclusion in brain disorders may bear even broader consequences. Many abused drugs are illegal in the western world and thus any individual dealing with these compounds (heroin, cocaine, amphetamines etc) is susceptible of imprisonment or, in the best cases, may be found guilty of use with consequent social stigma. However, if the addict is considered a patient he will “gain” a status, as anticipated earlier, free of moral connotations.

In conclusion, drug addiction is a medical condition attributed to an individual which has been assuming chemicals whose pharmacological effects produce a new brain equilibrium, called pathology. It can be documented in various ways (see above) thereby justifying the disease label. The organ afflicted is mainly (*but not only*) the brain and its fundamental byproduct (i.e., behavior) is severely diseased.

Denying its classification as a disease would be like precluding a patient his/her right to be cured.

## Reasoning

A final consideration: what strikes most in Levy’s account is that he spends many efforts in attempting to persuade the readership of what addiction “is not” whereas no effort is dedicated to describe what addiction actually “is.” At least in his mind. His statement: “these correlates are not sufficient for the person to have a disease in some accessible environments” represent, in my opinion, the “logic jump” in his flawed reasoning.

If we admit that the neural correlates of addiction are pathological (see above), and we admit that behavior stems from these neural correlates, it goes without saying that pathological neural correlates will yield pathological behavior (addiction). This conclusion can easily be inferred by invoking both deductive (facts are determined by combining existing statements) and inductive reasoning (facts are determined by repeated observations). Nowadays, we are well aware that both types of reasoning present different fallacies, but when both coincides “it is difficult to argue against facts, unfortunately ….”^1^

## Footnotes

^1^When Arvid Carlson presented to B. B. Brodie undisputable experimental results indicating that dopamine was a neurotransmitter in the brain of mammals, Brodie after opposing the idea for long, finally surrendered and, with a toothy grin erupting from his face, stated: “it is difficult to argue against facts, unfortunately ….” (G.L. Gessa, personal communication).
